# Is There Any Improvement in Image Quality in Obese Patients When Using a New X-ray Tube and Deep Learning Image Reconstruction in Coronary Computed Tomography Angiography?

**DOI:** 10.3390/life12091428

**Published:** 2022-09-13

**Authors:** Anne-Sofie Brunebjerg Pfeffer, Svea Deppe Mørup, Thomas Rueskov Andersen, Roda Abdulkadir Mohamed, Jess Lambrechtsen

**Affiliations:** 1 Cardiovascular Research Unit, Svendborg Hospital, Odense University Hospital, Baagøes Alle 15, 5700 Svendborg, Denmark; 2 Department of Clinical Research, University of Southern Denmark, Winsløwparken 19, 5000 Odense, Denmark; 3 Health Sciences Research Centre, UCL University College Lillebælt, Niels Bohrs Alle 1, 5230 Odense, Denmark; 4 Radiography & Diagnostic Imaging, School of Medicine, University College Dublin, D04 V1W8 Dublin, Ireland

**Keywords:** coronary computed tomography angiography (CCTA), coronary artery disease (CAD), deep learning image reconstruction (DLIR), image quality, diagnostic utility

## Abstract

Deep learning image reconstruction (DLIR) is a technique that should reduce noise and improve image quality. This study assessed the impact of using both higher tube currents as well as DLIR on the image quality and diagnostic accuracy. The study consisted of 51 symptomatic obese (BMI > 30 kg/m^2^) patients with low to moderate risk of coronary artery disease (CAD). All patients underwent coronary computed tomography angiography (CCTA) twice, first with the Revolution CT scanner and then with the upgraded Revolution Apex scanner with the ability to increase tube current. Images were reconstructed using ASiR-V 50% and DLIR. The image quality was evaluated by an observer using a Likert score and by ROI measurements in aorta and the myocardium. Image quality was significantly improved with the Revolution Apex scanner and reconstruction with DLIR resulting in an odds ratio of 1.23 (*p* = 0.017), and noise was reduced by 41%. A total of 88% of the image sets performed with Revolution Apex + DLIR were assessed as good enough for diagnosis compared to 69% of the image sets performed with Revolution Apex/CT + ASiR-V. In obese patients, the combination of higher tube current and DLIR significantly improves the subjective image quality and diagnostic utility and reduces noise.

## 1. Introduction

Ischemic heart disease is one of the leading causes of death and hospitalization [1]. The gold standard method in diagnosing stable coronary artery disease (CAD) is invasive coronary angiography (ICA). This is, however, an invasive procedure and therefore associated with risks of peri-procedural complications [2]. Coronary computed tomography angiography (CCTA) is an established non-invasive image modality for a rapid and accurate evaluation of the coronary arteries in patients with low to intermediate risk of stable CAD [3,4]. However, it is a challenge to obtain diagnostic image quality in obese patients due to increased noise from the excessive adipose tissue [3,5,6]. According to the World Health Organization (WHO), more than 650 million adults worldwide suffer from obesity, and the number is rising [7]. This represents a diagnostic challenge since obesity is also associated with an increased risk of CAD thus making clear visualization and evaluation of coronary arteries important in this particular group of patients [6,8]. 

Different techniques have been proposed to increase image quality in obese patients. This includes a higher tube current and an increase in tube potential which, theoretically, should improve image quality by reducing noise [9]. In this study, a new X-ray tube (Quantix 160 from GE Healthcare, Waukesha, WI, USA) has upgraded the Revolution CT scanner to a Revolution Apex (GE Healthcare, Waukesha, WI, USA) which can increase tube current up to 1300 mA for some kV settings and modulate radiation dose for each sampling [10]. The effect of higher tube currents is to increase the amount of the X-rays and to increase the signal reaching the detectors and hereby improve image quality.

Another promising technique in CT image reconstruction is the use of deep learning image reconstruction (DLIR), which is the newest reconstruction algorithm in CT [11]. DLIR has been developed to reduce noise and improve image quality without the need for a higher radiation dose [10,12,13]. The DLIR technique (DNN) is trained with high dose FBP images. The goal with DLIR is to reproduce good quality images from low dose examinations, by training the DNN to differentiate between signal and noise [14,15]. Few studies have already been performed on DLIR. The study by Solomon et al. has tested DLIR on phantoms and found reduced noise with the use of DLIR compared to conventional filtered back projection (FBP) [12]. Furthermore, Benz et al. have tested the diagnostic accuracy of DLIR in a clinical setting and compared it to adaptive statistical iterative reconstruction. Likewise, they found that DLIR significantly reduces noise in CCTA [11]. However, no studies have tested the combined effect of DLIR and the Revolution Apex in obese and very obese patients.

Therefore, the aim of this study is to evaluate possible improvements in image quality, diagnostic accuracy, and noise reduction in symptomatic obese and very obese patients with low to intermediate risk of stable CAD by using the Revolution Apex scanner and DLIR, respectively.

## 2. Materials and Methods

### 2.1. Patient Population

This study was approved by the local ethics committee, and informed consent was obtained from all patients. The study consecutively included 72 patients referred to a CCTA because of mild to moderate symptoms of CAD between December 2018 and July 2019. Out of these, 51 patients underwent CCTA twice. The follow-up scans were performed between October 2020 and November 2020. Originally, the follow-up was planned to be carried out approximately 6 months after the first examination, but the COVID-19 pandemic delayed the study. Patients were stratified by Body Mass Index (BMI) into two groups: (1) 21 patients with BMI 30–34.9 kg/m^2^ in the “obese” group and (2) 30 patients with BMI ≥ 35 kg/m^2^ in the “very obese” group. The inclusion criteria were age > 18 years, GFR > 45 mL/min, and heart rate (HR) between 50 and 85 beats per minute (BPM). Exclusion criteria were irregular heart rhythm, previous percutaneous coronary intervention (PCI), history of allergic reactions to contrast agents, and patients who had experienced significant cardiac events since the baseline CCTA. Significant cardiac events were defined as PCI or coronary artery bypass grafting (CABG).

On the day of the baseline CCTA, a patient examination and a structured interview provided information regarding risk factors: (1) BMI (height and weight), (2) blood pressure (BP), (3) smoking history (active, previous or nonsmoking), (4) diabetes mellitus (treatment with insulin, oral hypoglycemic agents, or non-pharmacological treatment), (5) family history of CAD, (6) treatment with anti-hypertensive drugs, and (7) treatment with lipid-lowering drugs. Furthermore, the indication of the CCTA was retrospectively obtained from each patient’s hospital record, and if “angina” was the indication, it was further specified (typical angina, atypical angina, unspecific chest pain, dyspnea, or other reasons).

On the day of the follow-up CCTA, the patients were interviewed regarding any significant changes in health status since their first CCTA. Significant changes were defined as: acute myocardial infarction (AMI), PCI, CABG, newly developed diabetes, and change in anti-hypertensive or lipid-lowering treatment. In addition, a new BMI was calculated.

### 2.2. Image Acquisition

The baseline CCTA examinations were performed on a 256-slice scanner (Revolution CT from GE Healthcare, Waukesha, WI, USA) with a gantry rotation time of 280 ms and a slice thickness of 0.625 mm. The tube voltages used were 100, 120, or 140 kV determined individually by the radiographers by evaluating each patient’s size. The automatic tube current modulation was set between 100 and 750 mA, and the scanner’s automatic tube current modulation (ATCM) selected the mA for all patients between 328 and 739 mA depending on patient size, and muscle and adipose tissue distribution.

The follow-up CCTA examinations were performed on the 256-slice CT scanner upgraded to the Revolution Apex with the new X-ray tube Quantix. Again, a rotation time of 280 ms, and a slice thickness of 0.625 mm were used, whereas the individual tube voltage from the patient’s baseline CCTA was set manually and therefore remained unchanged as well. ATCM automatically selected the tube current between 500 and 1300 mA (533–1080 mA).

All patients were prescribed 2 × 7.5 mg tablet of Ivabradine, 1 tablet the night before the scan, and 1 tablet in the morning of the CCTA. Moreover, all patients received 1 dose of sublingual nitroglycerin spray (0.4 mg/dose) 2–5 min before each CCTA in order to dilate the coronary arteries. HR was determined right before the scan. In case of a HR > 60 BPM, IV Metoprolol was given to lower the HR. Iodixanol contrast, Visipaque (GE Healthcare, Buckinghamshire, UK) with an iodine content of 320 mg iodine/mL was injected in a similar dose of 60–70 mL with an injection flow rate of 6–7 mL/s.

### 2.3. Image Reconstruction

All images were reconstructed with a standard kernel, a slice thickness of 0.625 mm, and 50% iterative reconstruction (ASiR-V) on the Revolution CT scanner. On the Revolution Apex scanner images were reconstructed with 50% ASiR-V as well as the DLIR algorithm TrueFidelity (GE Healthcare, Waukesha, WI, USA) with the level High. The DLIR kernel was added in the postprocessing process, after ASiR-V 50% reconstruction.

### 2.4. Subjective Image Quality Assessment

Image analysis was performed retrospectively. All image sets were assessed by a Level III CCTA certified cardiologist. Image quality was evaluated according to each of the quality criteria listed in Table 1. The assessment of criteria 1–6 was performed according to the 5-point Likert scale which grades the criteria into one of the 5 categories listed in Table 1 [16]. Additionally, the observer was asked to evaluate the diagnostic accuracy as either “diagnostic” or “non-diagnostic”. If the image sets were assessed as “non-diagnostic”, the observer selected between the following reasons for the artifact(s): A: *motions*, B: *noise*, C: *IV contrast*, and/or D: *calcium*. 

To test the reliability of the primary observer, another independent cardiologist assessed 11 randomly selected image sets under the same conditions. Both observers were asked to assess randomly picked CCTAs two times to detect intraobserver disagreements.

All image sets were randomized in ViewDEX 2.0 [17] and visually assessed by the observers who were blinded to clinical information, date of scan, and technical data. Only the standard series at the 75% phase was shown, and no further analysis was allowed for interpretation.

### 2.5. Objective Image Quality Assessment

Two independent reviewers performed all objective image quality measurements by placing regions of interest (ROI) on all image sets and by recording the mean Hounsfield unit (HU) value and corresponding noise measurement (standard deviation (SD)) value. Two ROIs were placed: (1) in the middle of the lumen of aorta (ROI_Ao_) with a size of 20 × 20 mm, and (2) in the interventricular septum in the myocardium (ROI_Myo_) with a mean size of 19 mm^2^ but modified based on anatomy. 

### 2.6. Radiation Dose 

Radiation dose was recorded by the volume CT dose index (CTDIvol), and the dose-length product (DLP) was generated from the dose report at the end of each scan.

### 2.7. Statistical Analyses

A power calculation with 20% improvement in Likert score with an 80% power and a 10% dropout rate estimates the need for 147 coronary arteries (47 patients).

The patient characteristics at baseline and follow-up, presented in Table 2, were calculated as means ± SD or simply presented as numbers [*n*] and as percentages of the total study population. Missing data were omitted, and in the specific case of the coronary artery calcium score (CAC), only patients with a known value in both the baseline and the follow-up scan were included to assure direct patient-to-patient comparison. CAC-scores were missing due to the local guidelines of not performing these in patients < 50 years. Comparisons between the groups were performed using a paired sample *t*-test.

The analyses of the subjective image quality were performed using an ordered logistic regression [proportional odds model] with the Likert scores [1,2,3,4,5] stratified by the CT scanner and reconstruction type (Revolution CT + ASiR-V, Revolution Apex + ASiR-V, and Revolution Apex + DLIR), and odds ratios (OR) were given. The same analysis model was performed with the “overall image quality” that was defined as the total sum of the Likert score from quality criteria 1–6. The diagnostic accuracy was calculated as the percentage of images assessed as “diagnostic” and “non-diagnostic”, respectively, for each of the three CT acquisition groups.

The analyses of the noise measurements were performed using linear regression with the ROI noise measurements (ROI_Ao_ and ROI_Myo_) with the “*Revolution CT + ASiR-V”* and the “*Revolution Apex + DLIR*” as dependent variables and the *”Revolution Apex + ASiR-V”* as the independent variable, and β-coefficients were calculated. Signal-to-noise ratios were calculated by dividing the Hounsfield units (HU) with the standard deviations (SD) and comparisons between the groups were performed using one way ANOVA for groups > 2 [18].

The intra- and inter-reader agreements were assessed by means of Bland–Altman plots and analyzed by the limits of agreement.

All statistical analyses were performed using © Stata/IC 16.0 (StataCorp LLC, College Station, TX, USA). A *p*-value of less than 0.05 was considered statistically significant.

## 3. Results

### 3.1. Patient Characteristics and Scan Parameters

Initially, a total of 72 patients met the inclusion criteria at the baseline scan and were included in the study. Of these, 21 patients were cut out afterwards due to the following exclusion criteria: irregular heart rhythm, HR > 85 BPM, PCI between baseline and follow-up scan, and/or technical issues with retrieving the correct image series needed for comparison. Additionally, some patients either did not show up or no longer wished to participate in the second CCTA partly due to the COVID-19 pandemic. Hence, the final study population consisted of 51 patients. In the time between the first and the second CCTA, four patients had changes in their cardiovascular pharmacological treatment, one patient had undergone a balloon angioplasty, and one patient had recently been diagnosed with type 2 diabetes. Furthermore, five patients had gained and three patients had lost more than 10 kg. Patient and CT acquisition characteristics are shown in Table 2.

### 3.2. Image Quality and Diagnostic Accuracy

**Figure 1:**
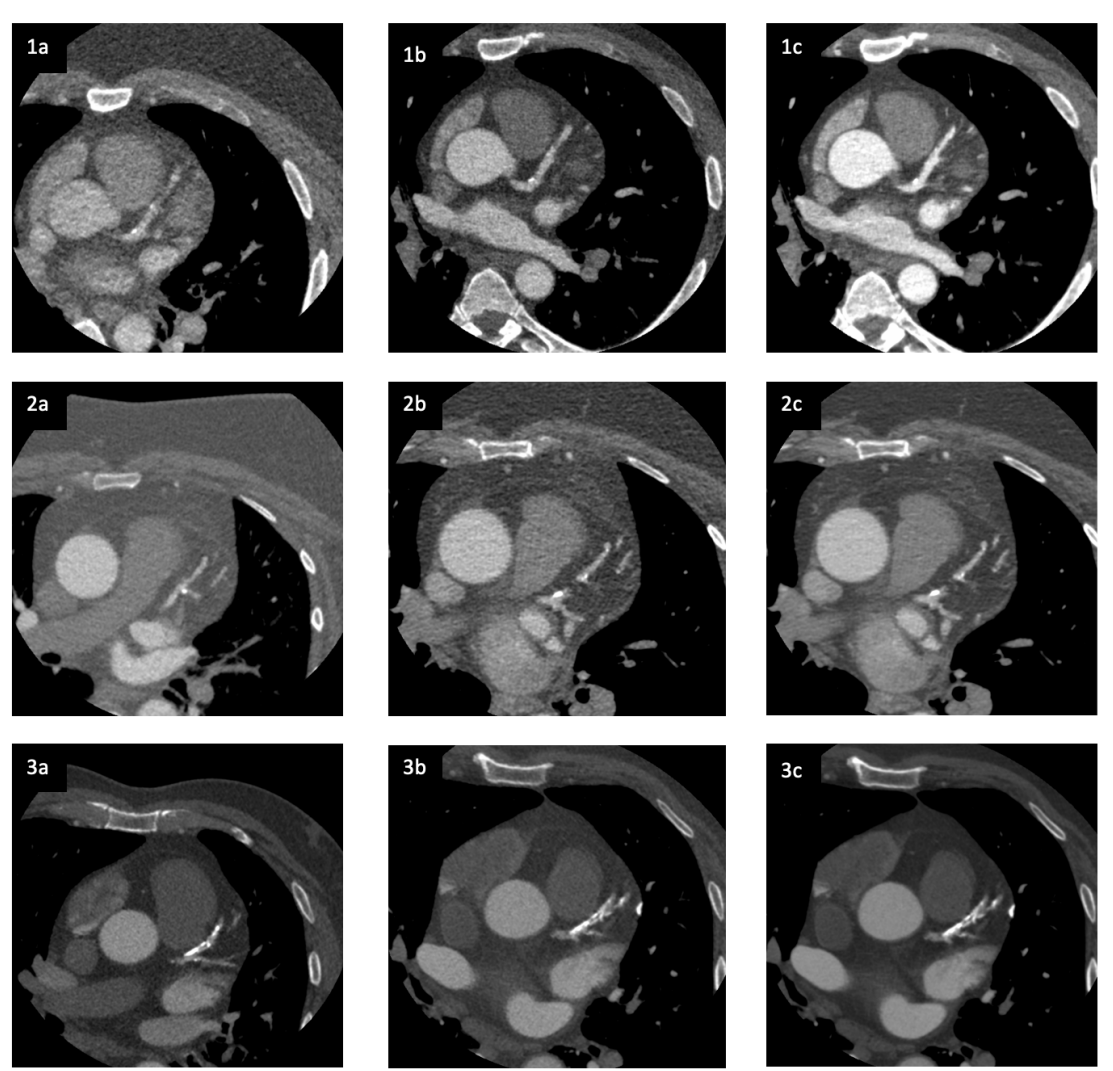
CCTA images from three different patients (1, 2, 3) illustrating (**a**) images performed with Revolution CT and reconstructed with ASiR-V, (**b**) images performed with Revolution Apex and reconstructed with ASiR-V, and (**c**) images performed with Revolution Apex and reconstructed with DLIR. Patient 1 (BMI 50), Patient 2 (BMI 40), and Patient 3 (BMI 32).

An overview of the image quality assessment is presented in Table 3. The overall image quality was significantly improved with Revolution Apex when images were reconstructed with DLIR (*p* = 0.017) compared to image sets performed with Revolution CT and conventional reconstruction with ASiR-V. For criterion (2) *Visually sharp delineation of the vessel wall of the left anterior descending artery (LAD)* (*p* = 0.001), criterion (5) *Visualization of the myocardial septum between the right and left ventricle* (*p* = 0.005), and criterion (6) *Homogeneity in the left/right ventricle* (*p* = 0.009) significant image quality improvements were found for Revolution Apex with DLIR. In the same criteria 2, 5, 6, and 7, no significant differences were found with Revolution Apex and conventional reconstruction with ASiR-V. Regardless of the CT scanner and the reconstruction method, no significant differences were found in criteria 1, 3, and 4 (sharp/clear delineation of the aortic wall, visually sharp delineation of the vessel wall of RCA and CX). Examples of the visual representation of the three different CCTA images from three of the patients is shown in Figure 1.

**Figure 2:**
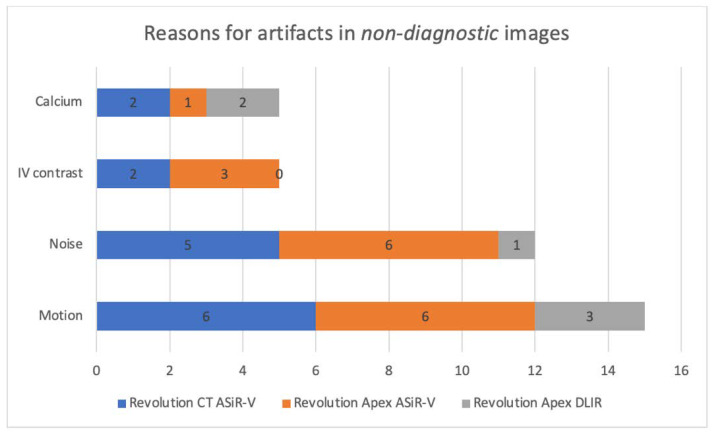
The selected reasons for artifacts in images assessed as “non-diagnostic”.

The diagnostic accuracy of the image sets performed with Revolution CT and ASiR-V was similar to those performed with Revolution Apex and ASiR-V with 69% of the images considered *diagnostic* and 31% *non-diagnostic*. However, with the use of Revolution Apex and DLIR, the diagnostic accuracy was improved with 88% of the images assessed as *diagnostic* and 12% as *non-diagnostic*. The reasons for the artifacts in *non-diagnostic* images are presented in Figure 2. It appears that the most frequently chosen reason for artifacts was *motion* closely followed by *noise*, except for the images performed with Revolution Apex and DLIR where only one of the artifacts in the image series was caused by noise.

### 3.3. Noise

The results of the ROI measurements are presented in Table 4. These results show a significant reduction in noise in the images performed with Revolution Apex DLIR compared to Revolution Apex ASiR-V. This was the case for both ROI_Ao_ (*p* ≤ 0.001) and ROI_Myo_ (*p* = 0.015). However, there were no significant changes in noise when using the Revolution Apex ASiR-V compared to Revolution CT ASiR-V. In Table 5, the mean Hounsfield units (HU), noise, and signal-to-noise ratio (SNR) are presented. Comparing the mean noise values for Revolution CT ASiR-V versus Revolution Apex ASiR-V and Revolution Apex DLIR, respectively, only findings for comparation with Revolution Apex DLIR were statistically significant in both ROI_Ao_ and ROI_Myo_. Furthermore, we also compared Revolution Apex ASiR-V and Revolution Apex DLIR and found a statistically significant difference. Thus, the Revolution Apex DLIR-modality reduced noise significantly in both ROI_Ao_ and ROI_Myo_. Examples of this noise reduction are visualized in Figure 1. Making the same comparision for signal-to-noise ratio, we found the same pattern with af statistically significant difference in signal-to-noise ratio when comparing Revolution CT ASiR-V and Revolution Apex DLIR as wells as Revolution Apex ASiR-V and Revolution Apex DLIR. These stastically significant findings were made both in the ROI_Ao_ and ROI_Myo_. There was no statistically significant difference in SNR between Revolution CT ASiR-V and Revolution Apex ASiR-V in neither aorta nor the myocardium.

### 3.4. Intra- and Interreader Agreement

**Figure 3:**
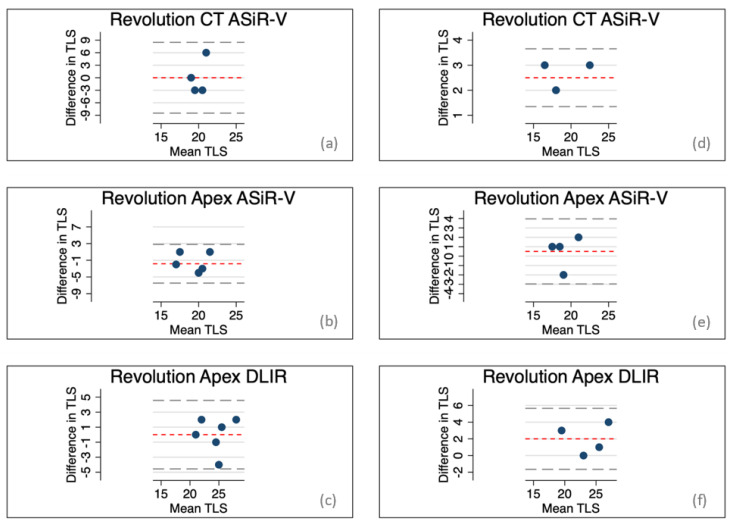
Bland–Altman plots with indication of mean differences and limits of agreement; (**a**–**c**) intraobserver agreement, (**d**–**f**) interobserver agreement. The agreements are determined by the total Likert scores.

The results of the intra- and interreader agreement are shown in Bland–Altman plots in Figure 3. The intrareader agreement showed quite large variations with the following mean differences ± SD (and 95% limits of agreement) in total Likert scores: 0.000 ± 4.243 (−8.315–8.315) with Revolution CT ASiR-V, −1.833 ± 2.317 (−6.374–2.707) with Revolution Apex ASiR-V, and 0.000 ± 2.280 (−4.469–4.469) with Revolution Apex DLIR. The interreader agreement did, as well, show quite large variations with the following mean differences ± SD (and 95% limits of agreement) in total Likert scores: 2.500 ± 0.577 (1.368–3.632) with Revolution CT ASiR-V, 0.500 ± 1.732 (−2.895–3.895) with Revolution Apex ASiR-V, and 2.000 ± 1.826 (−1.578–5.578) with Revolution Apex DLIR.

### 3.5. Radiation Dose

In average, the patients received a 18.35% higher radiation dose with the Revolution Apex scanner (mean DLP = 187 mGy*cm) compared to the Revolution CT (mean DLP = 158 mGy*cm).

## 4. Discussion

This study demonstrates that, compared to the Revolution CT and ASiR-V reconstruction, the Revolution Apex in combination with DLIR significantly increases image quality, reduces noise, and raises diagnostic image quality in obese and very obese patients. When eliminating DLIR and looking at the new Quantix 160 X-ray tube alone with conventional ASiR-V, no statistical improvements were detected–not in image quality, nor in diagnostic accuracy or noise reduction.

One of the hypotheses of this study was that the combined effect of the Revolution Apex with DLIR would result in significantly improved image quality. The study found improvements in image quality and an average noise reduction of 41%. This finding is consistent with the study from Benz et al. (2020) who have found a substantial reduction in image noise of up to 43% and an increase in image quality of up to 138% when reconstructing with DLIR compared to ASiR-V [11]. Another important finding of the present study is the increased diagnostic value of DLIR with 88% of the image sets performed with DLIR being approved for diagnostic use compared to only 69% with ASiR-V. This result differs from that of Benz et al. (2020) who found the diagnostic accuracy of DLIR to be equal to that of ASiR-V. However, the definition of “diagnostic quality” in our study is limited to a subjective assessment, while Benz et al. have tested the diagnostic accuracy by comparing the CCTA diagnoses with ICA. Our study is based on a larger study population, and the results could imply a greater diagnostic accuracy with the use of DLIR in obese and very obese patients. Moreover, Benz et al. only included patients with a high burden of CAD which makes the generalizability of their findings limited to this patient group. This could pose an issue since the use of CCTA as a stand-alone diagnostic tool is recommended only in patients with low to intermediate risk of CAD. The present study examines a group of patients with low to intermediate risk of CAD and is the first clinical trial to test DLIR in obese and very obese patients. Hereby, it contributes to filling an important gap in our present knowledge. With these results, it is fair to suggest that some of the results that Benz et al. found in high-risk patients regarding improved image quality would also apply to low to intermediate risk patients.

When analyzing the quality criteria individually, an improvement in image quality is seen in both criteria 5 and 6 regarding noise, while only one of the four criteria regarding spatial resolution, criteria 2, is significantly improved. This implicates that the positive effect of DLIR on image quality can mainly be ascribed to the noise reduction and less to the spatial resolution. This is further backed up by the results in Table 5 that show a significant reduction in noise with Revolution Apex + DLIR. These findings are consistent with that of Solomon et al. (2020) who have tested DLIR on different phantoms and found that, compared to FBP, DLIR demonstrates a strong noise magnitude reduction, while noise texture and high-contrast spatial resolution remain unchanged [12]. Even though the study design of these two studies has some important differences, the findings illustrate and support the same strengths and weaknesses of DLIR. This finding is further supported by the artifacts seen in the *non-diagnostic* images that are presented in Figure 2 where a decrease in the *noise* artifacts is seen. This supports the result that DLIR reduces noise and indicates that the noise reduction performed by DLIR has an impact on the diagnostic accuracy.

Contrary to expectations, the results of this study did not show any significant improvements in the image sets performed with Revolution Apex compared to conventional reconstruction with ASiR-V. Given the characteristics of the study population, which consists of only obese and very obese patients, it was hypothesized that an increase in tube current and radiation dose should result in improved image quality and noise reduction [9]. As seen in Table 2, both the tube current and the radiation dose have been increased as intended but without any significant effect on image quality and noise levels. These results could imply that the increase in tube current does not affect the visual image quality in obese or very obese patients when reconstructed with iterative reconstruction. However, another possible explanation could be the inclusion of both obese and very obese patients. Theoretically, image quality should decrease with increasing BMI due to increased noise from the excessive adipose tissue.

By increasing the tube current, the radiation dose was increased as well. Nevertheless, if this is followed by an improvement in image quality for this patient group, the benefits should compensate for the risks of a higher radiation dose. However, in this study there was no significant benefit from the increased radiation dose, and, consequently, it is reasonable to conclude that more studies regarding this topic are needed before recommending increased radiation doses in clinical practice. We hypothesize that it is better to use DLIR than to raise tube current to optimize image quality in obese patients when radiation dose is an issue.

This study has some limitations. First, the time between the two CCTAs was intended to be no longer than 6 months but was extended due to the previously mentioned issues. This has resulted in a maximum duration between the first and the second CCTA for almost 22 months for some patients. However, as the measured parameter in this study is the subjective image quality, the possible changes in cardiac health (such as increased CAC) during this period should not impact the comparability between the two CCTAs. Furthermore, each patient was interviewed and examined on the day of the second scan in order to detect any changes that might affect the comparison potential of the two CCTAs such as significant weight loss or gain. However, despite the individual changes in BMI observed in some of the patients, the two groups have shown to be similar and for that reason patients with change in BMI remained included in the study. As seen in Table 2, the CAC values have increased from the first CCTA to the second CCTA, and the change in CAC is statistically significant. The CAC, however, is expected to increase over time and should not implicate differences in the image quality assessments between baseline and follow-up especially concerning artefacts such as blooming, and we found no differences in blooming artefact from baseline to follow-up. A strength of this study is the direct comparison as the patients are compared to themselves. Another strength is the number of participants in this study, which increases its power and makes it the largest study regarding DLIR until today.

The relatively high number of non-diagnostic CCTAs is, of course, due to the previously mentioned limitations when scanning obese patients. In addition, the blinding method of the study with the restrictions for interpretation for the cardiologist of all available series of images further rises the proportion of non-diagnostic scans considerably.

The intra- and interobserver agreement were assessed and showed quite a large variation (Figure 3) which is not unusual and often seen in especially subjective image quality assessments. However, based on the primary observer’s level of experience the assessments have been considered as sufficient.

This study is based on obese and very obese patients with low to intermediate risk of CAD, which limits the generalizability of the results to this particular patient group. Yet, this group of patients was chosen because of the diagnostic challenge that patients with a high BMI represents when performing CCTA. Furthermore, if our study supported our initial hypotheses, our study would benefit this growing patient group. Future studies should examine other patient groups that represent a diagnostic challenge, e.g., patients with stents or patients with high CAC-scores. The DLIR used in this study, TrueFidelity, is available in both *Low, Medium*, and *High* levels, but in this study only the *High* level was included. Therefore, it could be interesting for future studies to include both Medium and Low levels and analyze the potential effect on spatial resolution.

## 5. Conclusions

In summary, reconstruction with DLIR in obese and very obese patients with a low to moderate risk of CAD has improved the perceived image quality and diagnostic accuracy by reducing noise levels compared to reconstruction with ASiR-V. The replacement of the previous X-ray tube with the new Quantix 160 and the increase in tube current did not result in any significant differences when reconstructing the image sets with the same reconstruction method (ASiR-V). More studies regarding both DLIR and the Quantix 160 X-ray tube are still needed.

## Figures and Tables

**Table 1:** Image quality criteria and Likert score.

Quality Criteria	
**1**	Sharp/clear delineation of the aortic wall
**2**	Visually sharp delineation of the vessel wall of LAD
**3**	Visually sharp delineation of the vessel wall of RCA
**4**	Visually sharp delineation of the vessel wall of CX
**5**	Visualization of the myocardial septum between the right and left ventricle
**6**	Homogeneity in the left/right ventricle
**Likert Score**	
**1**	High noise level, poor vessel definition
**2**	Considerable image noise, partially limited vessel wall delineation
**3**	Little image noise and good delineation of vessel borders
**4**	Good image quality, very little mage noise, and clear delineation of vessel walls
**5**	Excellent delineation of vessel walls, high attenuation in the vessel

Image quality criteria 1–6 and the 5-point Likert score were used to assess the image quality. LAD, left anterior descending artery; RCA, right coronary artery; CX, circumflex artery.

**Table 2:** Patient characteristics and scan parameters.

Characteristic	Revolution CT 18 December–19 July	Revolution Apex 20 October–20 November (*p*-Value)
**Age** [years], mean ± SD [range]	59 ± 11 [39–77]	60 ± 11 [41–79]
**Sex** [male/female], *n* [%]		
Male	26 [51]	26 [51]
Female	25 [49]	25 [49]
**BMI** [kg/m^2^]		
BMI, mean ± SD [range]	37 ± 5 [30–50]	37 ± 5 [27–49]
Obese [BMI 30–34.9], *n* [%]	21 [41]	20 [39]
Very obese [BMI ≥ 35], *n* [%]	30 [59]	31 [61]
**Cardiac risk factors**, *n* [%]		
Smoking [active or previous/non-smokers]	33/18 [65/35]	-
Diabetes mellitus	11 [22]	12 [24]
Family history of CAD	25 [49]	-
**Indication of CCTA**, *n* [%]		
Typical/atypical angina	17 [33]	-
Unspecified chest pain	27 [53]	-
Dyspnea	4 [8]	-
Other/unknown	3 [6]	-
**Medications**, *n* [%]		
Treatment with anti-hypertensive drugs	28 [55]	30 [59]
Treatment with lipid-lowering drugs	16 [31]	18 [35]
**Coronary artery calcium score [CAC]**, mean ± SD	199 ± 497	239 ± 524
**Heart rate during CT acquisition**, mean ± SD	57 ± 7	58 ± 7
**Tube current [mA]**, mean ± SD	619 ± 80	828 ± 138 (<0.001)
**Dose length product [DLP]** [mGy*cm], mean ± SD	158 ± 96	187 ± 101 (0.022)

Patient characteristics and scan parameters at baseline scan [Revolution CT] and follow-up scan [Revolution Apex]. *p*-values are only provided when significant.

**Table 3:** Results for each image quality criteria.

Image Quality Criteria	CT Scanner and *Reconstruction Method*	Odds Ratio [95% CI]	*p*-Value
1. Sharp/clear delineation of the aortic wall	Revolution CT *ASiR-V* [ref.]		
	Revolution Apex *ASiR-V*	1.86 [0.66–5.21]	0.24
	Revolution Apex *DLIR*	1.51 [0.71–3.24]	0.29
2. Visually sharp delineation of the vessel wall of LAD	Revolution CT *ASiR-V* [ref.]		
	Revolution Apex *ASiR-V*	0.76 [0.31–1.85]	0.55
	**Revolution Apex *DLIR***	**3.30 [1.64–6.54]**	**0.001**
3. Visually sharp delineation of the vessel wall of RCA	Revolution CT *ASiR-V* [ref.]		
	Revolution Apex *ASiR-V*	1.05 [0.44–2.46]	0.92
	Revolution Apex *DLIR*	1.76 [0.85–3.64]	0.13
4. Visually sharp delineation of the vessel wall of CX	Revolution CT ASiR-V [ref.]		
	Revolution Apex *ASiR-V*	1.96 [0.78–4.96]	0.15
	Revolution Apex *DLIR*	1.31 [0.63–2.72]	0.48
5. Visualization of the myocardial septum between the right and left ventricle	Revolution CT ASiR-V [ref.]		
	Revolution Apex ASiR-V	0.66 [0.20–2.14]	0.49
	**Revolution Apex DLIR**	**4.82 [1.60–14.56]**	**0.005**
6. Homogeneity in the left/right ventricle	Revolution CT ASiR-V [ref.]		
	Revolution Apex *ASiR-V*	0.66 [0.20–2.14]	0.49
	**Revolution Apex *DLIR***	**3.26 [1.34–7.91]**	**0.009**
7. Overall image quality	Revolution CT ASiR-V [ref.]		
	Revolution Apex ASiR-V	0.999 [0.80–1.25]	0.995
	**Revolution Apex DLIR**	**1.23 [1.04–1.46]**	**0.017**

Results for each image quality criteria 1–6, assessed with the Likert score, and the overall image quality [criterion 7] as a result of the total Likert score. Image series performed with Revolution CT and reconstructed with ASiR-V was the reference scan and reconstruction method. Criteria 1–4 are connected to the spatial resolution and criteria 5–6 to the noise.

**Table 4:** Results of the regions of interest (ROI).

ROI	CT Scanner and Reconstruction Method	β-Coefficient	95% CI	*p*-Value
**Aorta**	Revolution CT *ASiR-V*	0.986	−0.996–2.644	0.371
	**Revolution Apex *DLIR***	**0.658**	**0.454–0.863**	**<0.001**
**Myocardium**	Revolution CT *ASiR-V*	0.913	−3.562–1.002	0.269
	**Revolution Apex *DLIR***	**0.329**	**0.068–0.590**	**0.015**

Results of the regions of interest (ROI) in aorta and the myocardium. Image series performed with [Revolution Apex and ASiR-V] were used as the predictor variable.

**Table 5:** Results of the objective ROI-measurements.

ROI	CT Scanner and Reconstruction Method	Mean HU	Mean Noise (±SD), (*p*-Value)	SNR (±SD), (*p*-Value)
**Aorta**	Revolution CT *ASiR-V*	423.3	39.5 (±4.6)	11.1 (±2.1)
	Revolution Apex ASiR-V	478.0	40.3 (±5.0)	10.8 (±2.6)
	Revolution Apex *DLIR*	478.5	**21.7 (±4.6), (<0.0001)**	**21.4 (** **±6.1), (0.001)**
**Myocardium**	Revolution CT *ASiR-V*	70.5	36.6 (±4.7)	2.0 (±0.6)
	Revolution Apex *ASiR-V*	78.6	37.9 (±6.9)	2.0 (±0.4)
	Revolution Apex *DLIR*	78.7	**23.3 (±4.9), (<0.0001)**	**3.3 (** **±1.0), (0.001)**

Results of the objective ROI-measurements including the mean Hounsfield units (HU), mean noise (SD), and the signal-to-noise-ratio (SNR). The *p*-values are only presented if statistically significant.

## Data Availability

The data that support the findings of this study are available on request from the corresponding author, [A.-S.B.P.]. The data are not publicly available due to its containing of information that could compromise the privacy of research participants.

## Abbreviations

CAD	coronary artery disease
ICA	invasive coronary angiography
CCTA	coronary computed tomography angiography
WHO	World Health Organization
DLIR	deep learning image reconstruction
BMI	body mass index
HR	heart rate
BPM	beats per minute
PCI	percutaneous coronary intervention
CABG	coronary artery bypass grafting
BP	blood pressure
AMI	acute myocardial infarction
ATCM	automatic tube current modulation
ASiR-V	adaptive statistical iterative reconstruction–veo
ROI	region of interest
HU	Hounsfield unit
SD	Standard deviation
CTDI	computed tomography dose index
CAC	coronary artery calcium
OR	odds ratio
LAD	left anterior descending (artery)
RCA	right coronary artery
CX	circumflex (artery)
DLP	dose length product

## References

[1] World Health Organisation The Top 10 Causes of Death 2020. https://www.who.int/news-room/fact-sheets/detail/the-top-10-causes-of-death.

[2] Segev O.L., Gaspar T., Halon D.A., Peled N., Domachevsky L., Lewis B.S., Rubinshtein R. (2012). Image quality in obese patients undergoing 256-row computed tomography coronary angiography. Int. J. Cardiovasc. Imaging.

[3] Zimmerman S.L., Kral B.G., Fishman E.K. (2014). Diagnostic quality of dual-source coronary CT examinations performed without heart rate control: Importance of obesity and heart rate on image quality. J. Comput. Assist. Tomogr..

[4] Montalescot G., Sechtem U., Achenbach S., Andreotti F., Arden C., Budaj A., Bugiardini R., Crea F., Cuisset T., Di Mario C. (2013). 2013 ESC guidelines on the management of stable coronary artery disease: The Task Force on the management of stable coronary artery disease of the European Society of Cardiology. Eur. Heart J..

[5] Ghekiere O., Salgado R., Buls N., Leiner T., Mancini I., Vanhoenacker P., Dendale P., Nchimi A. (2017). Image quality in coronary CT angiography: Challenges and technical solutions. Br. J. Radiol..

[6] Lee A.M., Engel L.C., Hui G.C., Liew G., Ferencik M., Sidhu M.S., Hoffmann U., Ghoshhajra B.B. (2014). Coronary computed tomography angiography at 140 kV versus 120 kV: Assessment of image quality and radiation exposure in overweight and moderately obese patients. Acta Radiol..

[7] World Health Organisation Obesity and Overweight 2020. https://www.who.int/news-room/fact-sheets/detail/obesity-and-overweight.

[8] Lu Y., Hajifathalian K., Ezzati M., Woodward M., Rimm E.B., Danaei G. (2014). Metabolic mediators of the effects of body-mass index, overweight, and obesity on coronary heart disease and stroke: A pooled analysis of 97 prospective cohorts with 1.8 million participants. Lancet.

[9] Seeram E. (2016). Computed Tomography: Physical Principles, Clinical Applications, and Quality Control.

[10] Thibault J., Utschig M. (2020). Revolution Apex with QuantixTM 160 [White Paper]. https://www.gehealthcare.co.uk/products/computed-tomography/revolution-apex.

[11] Benz D.C., Benetos G., Rampidis G., von Felten E., Bakula A., Sustar A., Kudura K., Messerli M., Fuchs T.A., Gebhard C. (2020). Validation of deep-learning image reconstruction for coronary computed tomography angiography: Impact on noise, image quality and diagnostic accuracy. J. Cardiovasc. Comput. Tomogr..

[12] Solomon J., Lyu P., Marin D., Samei E. (2020). Noise and spatial resolution properties of a commercially available deep learning-based CT reconstruction algorithm. Med. Phys..

[13] Hsieh J., Liu E., Nett B., Tang J., Thibault J., Sahney S. (2020). A New Era of Image Reconstruction: TrueFidelityTM Technical White Paper on Deep Learning Image Reconstruction [White Paper]. https://www.gehealthcare.com/-/jssmedia/040dd213fa89463287155151fdb01922.pdf.

[14] Zhang Z., Seeram E. (2020). The use of artificial intelligence in computed tomography image reconstruction—A literature review. J. Med. Imaging Radiat. Sci..

[15] Arndt C., Güttler F., Heinrich A., Bürckenmeyer F., Diamantis I., Teichgräber U. (2021). Deep Learning CT Image Reconstruction in Clinical Practice. Fortschr. Röntgenstr..

[16] Seppelt D., Kolb C., Kühn J.P., Speiser U., Radosa C.G., Hoberück S., Hoffmann R.T., Platzek I. (2019). Comparison of sequential and high-pitch-spiral coronary CT-angiography: Image quality and radiation exposure. Int. J. Cardiovasc. Imaging.

[17] Håkansson M., Svensson S., Zachrisson S., Svalkvist A., Båth M., Månsson L.G. (2010). VIEWDEX: An efficient and easy-to-use software for observer performance studies. Radiat. Prot. Dosim..

[18] Sookpeng S., Martin C.J., Butdee C. (2019). The investigation of dose and image quality of chest computed tomography using different combinations of noise index and adaptive statistic iterative reconstruction level. Indian J. Radiol. Imaging.

